# Output-Specific Adaptation of Habenula-Midbrain Excitatory Synapses During Cocaine Withdrawal

**DOI:** 10.3389/fnsyn.2021.643138

**Published:** 2021-03-31

**Authors:** Joseph Clerke, Patricia Preston-Ferrer, Ioannis S. Zouridis, Audrey Tissot, Laura Batti, Fabian F. Voigt, Stephane Pagès, Andrea Burgalossi, Manuel Mameli

**Affiliations:** ^1^The Department of Fundamental Neuroscience, The University of Lausanne, Lausanne, Switzerland; ^2^Institute of Neurobiology, University of Tübingen, Tübingen, Germany; ^3^Werner-Reichardt Centre for Integrative Neuroscience, Tübingen, Germany; ^4^International Max Planck Research School, Graduate Training Centre of Neuroscience, University of Tübingen, Tübingen, Germany; ^5^The Wyss Center for Bio and Neuroengineering, Geneva, Switzerland; ^6^Brain Research Institute, University of Zurich, Zurich, Switzerland; ^7^Neuroscience Center Zurich, University of Zurich & ETH Zurich, Zurich, Switzerland; ^8^INSERM UMR-S 839, Paris, France

**Keywords:** ventral tegmental area, lateral habenula, cocaine, synaptic plasticity, glutamatergic transmission

## Abstract

Projections from the lateral habenula (LHb) control ventral tegmental area (VTA) neuronal populations’ activity and both nuclei shape the pathological behaviors emerging during cocaine withdrawal. However, it is unknown whether cocaine withdrawal modulates LHb neurotransmission onto subsets of VTA neurons that are part of distinct neuronal circuits. Here we show that, in mice, cocaine withdrawal, drives discrete and opposing synaptic adaptations at LHb inputs onto VTA neurons defined by their output synaptic connectivity. LHb axons innervate the medial aspect of VTA, release glutamate and synapse on to dopamine and non-dopamine neuronal populations. VTA neurons receiving LHb inputs project their axons to medial prefrontal cortex (mPFC), nucleus accumbens (NAc), and lateral hypothalamus (LH). While cocaine withdrawal increases glutamate release from LHb onto VTA-mPFC projectors, it reduces presynaptic release onto VTA-NAc projectors, leaving LHb synapses onto VTA-to-LH unaffected. Altogether, cocaine withdrawal promotes distinct adaptations at identified LHb-to-VTA circuits, which provide a framework for understanding the circuit basis of the negative states emerging during abstinence of drug intake.

## Introduction

The functional role of distinct ventral tegmental area (VTA) neuronal populations is a matter of long-standing interest as through the innervation of their target structures, these cells underlie core components of rewarding and aversive behaviors as well as of neuropsychiatric disorders including drug addiction (Nestler and Lüscher, 2019). To mediate this, the VTA relies on neuronal populations that are heterogeneous in their anatomical location, and especially in the targets to which they project (Lammel et al., 2014). Indeed, VTA neurons projecting to the medial prefrontal cortex (mPFC), the nucleus accumbens (NAc) and the lateral hypothalamus (LH) are crucial for motivated behaviors and the related pathological states (Lammel et al., 2014).

VTA neurons receive, among others, glutamatergic innervation from the epithalamic lateral habenula (LHb) (Omelchenko et al., 2009). LHb axons onto the midbrain exert profound control of neuronal activity, by directly exciting some VTA cells as well as reducing the activity of other VTA neurons. As a consequence this control leads to the expression of aversive behaviors (Matsumoto and Hikosaka, 2007; Lammel et al., 2012). Drug withdrawal increases excitatory synaptic efficacy and neuronal activity within the LHb leading to the emergence of drug-driven depressive states (Meye et al., 2015, 2017). Along with this, cocaine withdrawal also produces a wealth of synaptic adaptations and changes in excitability of VTA neuronal populations (Chen et al., 2008; Ishikawa et al., 2013). However, little is known about the repercussions cocaine withdrawal may have on LHb-dependent control of VTA neuronal populations, especially in light of the circuits in which they are embedded.

By employing brain clarification, and viral-based anatomical tracing in combination with *ex vivo* electrophysiology, here we define functional differences between identified LHb-to-VTA output circuits undergoing synaptic adaptations during cocaine withdrawal.

## Materials and Methods

### Animals and Cocaine Treatment

4–10 week old male wild-type mice (C57/BL6J) were group-housed (five per cage) with food and water *ad libitum* on a 12–12 h light cycle (lights on at 7:00). All procedures aimed to fulfil the criterion of the 3Rs and were approved by the Veterinary Offices of Vaud (Switzerland; license VD3172.1). Mice received five consecutive daily intraperitoneal (i.p.) cocaine (20 mg/kg) or saline injections (saline and cocaine-treated animals were housed together) and were sacrificed for electrophysiology experiments 10–15 days after the last injection.

### Stereotactic Injections

4–5 week old male mice were anesthetized with ketamine (150 mg/kg)/xylazine (100 mg/kg) and placed on a stereotactic frame (Kopf, Germany). Bilateral injections (200–300 nl) obtained through a glass pipette were performed at a rate of approximately 100 nl min^–1^. The injection pipette was slowly retracted from the brain 10 min following injection. The following coordinates (in mm relative to bregma and skull surface) were used for injections: lateral habenula [LHb: Anterior-Posterior (AP) −1.4, Media-Lateral (ML) ± 0.45 mm, Dorsal-Ventral (DV) −3.1]; medial prefrontal cortex (mPFC: AP 1.9, ML ± 0.37, −2.2); nucleus accumbens (NAc: AP 1.6, ML ± 0.5 mm, DV −4.5); lateral hypothalamus (LH: AP −1.34, ML ± 0.9, DV −5.08); medial VTA (mVTA: AP −2.7, ML ± 0.3, DV −4.6). A minimum of 5 days recovery were allowed after viral infusion prior to treatment with cocaine or saline. Viral constructs used in the study: rAAV8-hSyn-Chrimson-tdTomato (rAAV8-tdTomato; University of Pennsylvania viral vector core; titer: 7 × 10^12^ gc/ml). HSV-pEF1α-mCherry (HSV-mCherry; R Neve, Viral Gene Transfer Core Facility of the Massachusetts Institute of Technology; titer: 3.5 × 10^9^ gc/ml). rAAV1-hSyn-Cre-WPRE-hGH (rAAV1:ht-Cre; University of Pennsylvania viral vector core; titer:2.5 × 10^13^ gc/ml). rAAV8-hSyn-ChR2 (H134R)-GFP (rAAV8-ChR2-GFP; Addgene 58880; titer: 3.3 × 10^13^ gc/ml). rAAV8-HSyn-dLox-EGFP-dLox-WPRE-HGHp(A) (rAAV8-Flex-EGFP; Viral Vector Facility UZH, titer: 6.4 × 10^12^ gc/ml). rAAV2.5-hSyn-mRuby-T2A-Synaptophysin-eGFP (rAAV5-mRuby-T2A-Synaptophysin-eGFP; B.K. Lim, UCSD; titer: 10^13^ gc/ml). Injection sites were carefully examined during all electrophysiology and anatomical experiments, and only animals with correct injections were included in the study. For injections of viruses leaving no visible fluorescent trace within the injections site (HSV-mCherry; rAAV1:ht-Cre) a small amount of diluted red retrobeads (Lumaflor) were concurrently injected to enable identification *post hoc* of injection site.

### *Ex vivo* Electrophysiology

7–9 week old male mice were anesthetized with an injection of ketamine (150 mg/kg)/xylazine (100 mg/kg) for preparation of acute brain slices as previously published (Li et al., 2011; Meye et al., 2015). Following sacrifice, brains were rapidly extracted and placed in ice cold 95% O^2^/5% CO^2^ – equilibrated bubbled slicing solution containing (pH 7.4, 298-302 mOsm, in mM): 110 choline chloride, 25 glucose, 25 NaHCO3, 7 MgCl2, 11.6 ascorbic acid, 3.1 sodium pyruvate, 2.5 KCl, 1.25 NaH2PO4, and 0.5 CaCl2. Horizontal slices (250 μm) containing the VTA were prepared and transferred for 5 min to heated solution (34°C) of identical composition before they were stored at room temperature in 95% O^2^/5% CO^2^ -equilibrated artificial cerebrospinal fluid (aCSF, pH 7.4, 310 mOsm) containing (in mM): 124 NaCl, 26.2 NaHCO3, 11 glucose, 2.5 KCl, 2.5 CaCl2,1.3 MgCl2, and 1 NaH2PO4. Recordings were conducted using an Olympus-BX51 microscope (Olympus) at 31°C (flow rate of 2.5 ml min^–1^). Borosilicate glass pipettes (2.7–4 MΩ; Phymep) were used for whole-cell patch-clamp experiments (all in voltage-clamp configuration). Currents were amplified, filtered at 5 kHz and digitized at 20 kHz (Multiclamp 200B; Molecular Devices). Data were acquired using Igor Pro with NIDAQ tools (Wavemetrics). Recordings were discarded if the access resistance (monitored using a −4 mV step per 10 s) increased by more than 20%. Spontaneous EPSCs were recorded at −60 mV whereas presynaptic stimulation trains were recorded at −50 mV. Both of these experiments were conducted in the presence of picrotoxin (100 μM, Hello Bio) and APV (100 μM, Hello Bio). The internal solution used to measure spontaneous EPSCs and presynaptic release probability contained (in mM): 140mM potassium gluconate, 4 mM NaCl, 2mM MgCl_2_, 1.1mM EGTA, 5mM HEPES, 2mM Na2ATP, 5mM sodium creatine phosphate, and 0.6mM Na3GTP (pH 7.3 with KOH; 290–300 mOsm; Labouèbe et al., 2007). Light pulses (490 nm, 1–5 ms) for opto-stimulation of LHb nerve terminals were delivered with a LED (CoolLed, United Kingdom) illumination system. To assess LHb terminal presynaptic release properties, trains of AMPAR-EPSCs were evoked (10 light pulses at 10 and 20 Hz). The amplitudes of EPSC trains were normalized to the amplitude of the first pulse. The internal solution used to measure AMPAR/NMDAR ratios contained (in mM): 130 CsCl, 4 NaCl, 2 MgCl2, 1.1 ethylene glycol tetraacetic acid (EGTA), 5 HEPES buffer, 2 ATP-Na2, 5 sodium creatine-phosphate, 0.6 GTP-Na3, and 0.1 spermine (pH 7.3 with KOH; 290–300 mOsm; Bellone and Lüscher, 2006). To determine AMPA/NMDA ratios at LHb-to-VTA synapses, firstly, a mixed AMPA and NMDA current was evoked using a single light pulse at + 40 mV (in the presence of picrotoxin). Next, distinct AMPA and NMDA components were pharmacologically isolated by adding APV in the aCSF and by subsequent identification of the individual currents via digital subtraction (Mameli et al., 2009; Maroteaux and Mameli, 2012). For output-specific experiments, retrogradely labeled and fluorescently identified (mCherry +) VTA neurons were recorded from.

### Histology and Immunofluorescence

Male mice were anesthetized and transcardially perfused with cold PBS followed by 4% paraformaldehyde (PFA) in PBS. The brains were carefully extracted, post-fixed in 4% PFA in PBS for 24 h and then incubated in PBS until they sank prior to sectioning. For mice undergoing viral infusion with rAAV1:ht-Cre and rAAV8-Flex-EGFP mice were perfused 3 weeks following viral infusion and coronal slices (50 μm) were prepared using a vibratome and stored in PBS containing 0.1%NaAz for future analyses. For immunofluorescence, slices were incubated for 2 h in blocking buffer [10%NGS (normal goat serum) in PBS] and then incubated for 24 h at 4°C with the primary antibody solution [rabbit anti-TH: ab112, abcam (1:500) in a carrier solution containing 3% NGS in PBS]. After extensive rinses with PBS, slices were incubated in the secondary antibody solution goat anti-rabbit IgG-conjugated Alexa 647, Invitrogen (1:500 in carrier solution), for 2 h at room temperature. The slices were then extensively rinsed, mounted on glass slides with Vectashield Antifade Mounting Medium with DAPI (Vectorlabs) and coverslipped. For synaptophysin experiments, horizontal sections (50 μm) were prepared using a vibratome for preferential visualization of the VTA. Confocal microscopy was performed using a TCS-SP5 (Leica) Laser Scanning System with ×10 and ×20 dry objectives. Images were acquired using the same parameters for all the samples within each experiment, then processed and analyzed using the software ImageJ. Proportions of LHb receiving cells co-stained with TH were determined using the cell counter plugin from an average of 5 slices per mouse covering the rostrocaudal extent of the VTA. For calculation of relative arbitrary fluorescence of synaptophysin punctae, green signal intensity was normalized against a region containing no green fluorescence (background) using the formula: [(Signal-background/Signal + background)/Area)]. For synaptophysin experiments, four slices distributed in the dorsoventral axis were analyzed per animal. Example images of rAAV8-ChR2-GFP injected mice, HSV-mCherry injected (VTA output) mice, and LHb-receiving VTA projections were taken with an epifluorescence microscope with ×2.5, ×5, and ×10 objectives (AxioVision, Zeiss).

### *In vivo* Juxtacellular Labeling

*In vivo* juxtacellular labeling was performed on anesthetized male C57BL/6J mice (>6 weeks old; Charles River) as described previously (Diamantaki et al., 2018). Briefly, under ketamine/xylazine anesthesia, a craniotomy was performed at the coordinates for targeting the LHb (1.3–1.6 mm posterior and 0.6 mm lateral from bregma). Glass electrodes with resistance 5–7 MΩ were filled with 1.5–2% Neurobiotin (Vector Laboratories) in Ringer’s solution containing (in mM): 135 NaCl, 5.4 KCl, 5 HEPES, 1.8 CaCl_2_, and 1 MgCl_2_ or Intracellular solution containing (in mM): 135 K-gluconate, 10 HEPES, 10 Na_2_-phosphocreatine, 4 KCl, 4 MgATP, and 0.3 Na_3_GTP. Osmolarity was adjusted to 280–310 mOsm. Juxtacellular labeling was performed according to standard procedures with 200 ms-long squared current pulses. The juxtacellular voltage signal was acquired via an ELC-03XS amplifier (NPI Electronic), sampled at 20 kHz by a Power1401-3 analog-to-digital interface under the control of Spike2 software (CED, Cambridge, United Kingdom).

For histological processing, animals were euthanized with an overdose of pentobarbital and perfused transcardially with 0.1 M PBS followed by a 4% paraformaldehyde solution. Brains were sliced on a vibratome (VT1200S; Leica) to obtain 70 μm-thick coronal sections. To reveal the morphology of juxtacellularly labeled cells (i.e., filled with neurobiotin), brain slices were processed with Streptavidin-546 (Life Technologies) as described previously (Diamantaki et al., 2018). Fluorescent images were acquired by epifluorescence microscopy (Axio imager; Zeiss). After fluorescence images were acquired, the neurobiotin staining was converted into a dark DAB reaction product followed by Ni_2_+-DAB enhancement protocol.

### CLARITY – Sample Preparation and Lightsheet Imaging

Tissue used in CLARITY experiments underwent viral injection (unilateral injection of LHb with 250nl rAAV8-tdTomato) and perfusion/brain extraction as described above prior to being shipped to the Wyss Center, Geneva. Brains were clarified following the CLARITY protocol (Chung and Deisseroth, 2013), using a X-CLARITY^TM^ system. Brains were immersed in a refractive index matching solution (RIMS) containing Histodenz (Sigma Aldrich) for at least 24 h before being imaged. Imaging was performed using a mesoSPIM (Voigt et al., 2019) instrument at Wyss Center, Geneva. The microscope consists of a dual-sided excitation path using a fiber-coupled multiline laser combiner (405, 488, 561, and 647 nm, Toptica MLE) and a detection path comprising an Olympus MVX-10 zoom macroscope with a 1× objective (Olympus MVPLAPO 1×), a filter wheel (Ludl 96A350), and a scientific CMOS (sCMOS) camera (Hamamatsu Orca Flash 4.0 V3, 2048 × 2048 pixels). The excitation paths also contain galvo scanners for light-sheet generation and reduction of shadow artifacts due to absorption of the light-sheet. In addition, the beam waist is scanned using electrically tunable lenses (ETL, Optotune EL-16-40-5D-TC-L) synchronized with the rolling shutter of the sCMOS camera. This axially scanned light-sheet mode (ASLM) leads to a uniform axial resolution across the field-of-view (FOV) of 6.5 μm. Image acquisition is done using custom software written in Python. Z-stacks were acquired with a zoom set at 0.8X and 2X, at 5 and 3 μm spacing, respectively, resulting in an in-plane pixel size of 8.2 μm × 8.2 μm and 3.26 μm × 3.26 μm, respectively (2048 × 2048 pixels). Excitation wavelength of the tdTomato was set at 561 nm with an emission filter LP 561 nm longpass filter (BrightLine HC, AHF).

### Quantification and Statistical Analysis

Online and offline analysis for presynaptic trains data were performed using Igor Pro-6 (Wavemetrics, United States). sEPSCs recordings were manually analyzed offline using Minianalysis (Synaptosoft Inc, United States). Sample size was predetermined on the basis of published studies and in-house expertise. Animals were randomly assigned to experimental groups. Compiled data for sEPSC and AMPA/NMDA recordings are reported and represented as violin plots (median and quartiles) with single data points (cells) plotted. Compiled data for presynaptic LHb stimulation trains are represented as mean ± SEM of normalized current amplitude for each pulse. Datasets were screened for outliers using a ROUT test; outliers removed are stated in figure legends. Data collection and analyses were not performed blinded to the conditions of the experiments. Data distribution was tested for normality. When applicable, statistical tests were two-way ANOVAs or unpaired *t*-test. In case of not-normally distributed data, we used the Mann-Whitney or Kolmogorov-Smirnov tests. Testing was always performed two-tailed with *α* = 0.05.

## Results

### Midbrain Innervation by LHb Axonal Projections

**FIGURE 1:**
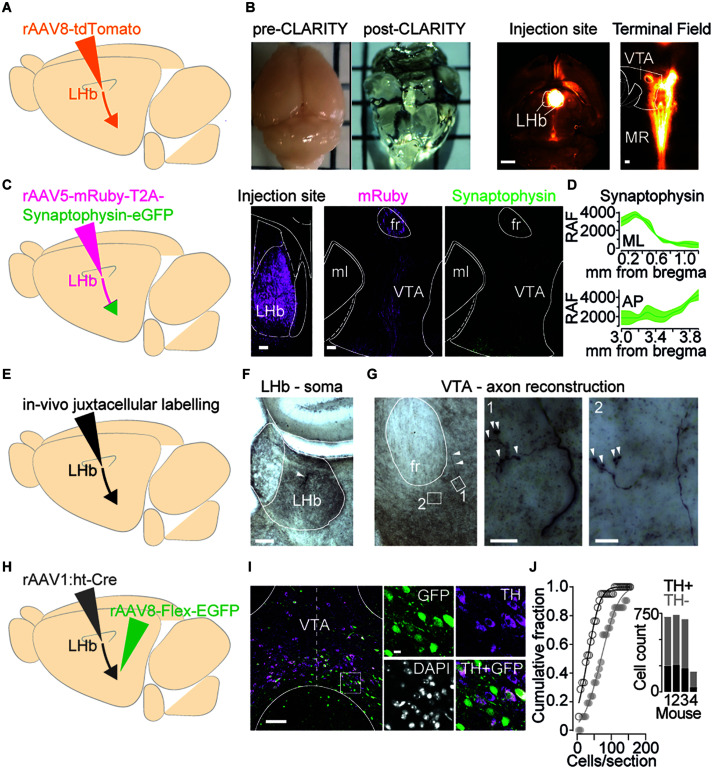
Axons from the LHb innervate midbrain cell populations. **(A)** Viral strategy to visualize wholebrain projectome of LHb. **(B)** Left: Examples of a mouse brain before and after clarification treatment. Right: Representative images (horizontal plane) of LHb viral injection site (1 mm) and tdTomato positive terminals targeting the VTA and Median Raphe Nucleus (MRN) (100 μm). **(C)** Left: Viral strategy to identify LHb axon terminals in the midbrain. Right: images (horizontal plane, 100 μm, 10×) left to right: injection site in the LHb; mRuby positive axons in the VTA; Synaptophysin-EGFP positive terminals in the VTA. **(D)** Quantification in Relative Arbitrary Fluorescent (RAF) Units of Synaptophysin expression throughout the mediolateral (ML, top) and Anterior-Posterior (AP, bottom) extent of the VTA (n_hemispheres/mice_ = 3/2) (mean ± SEM). **(E)** Strategy to identify individual LHb neuron axon terminal boutons in the VTA. **(F)** Coronal DAB-stained section showing a single LHb neuron (white arrowhead) juxtacellularly labeled *in vivo* (scale bar, 100 μm). **(G)** Left: DAB-stained section showing a single LHb axon (white arrowheads) emerging from fr into VTA (scale bar, 100 μm). Center and right, Maximum intensity z-stack projections of close-up magnifications (1 and 2) showing synaptic boutons within the VTA (white arrowheads) (scale bars, 10 μm). **(H)** Viral strategy to label LHb receiving neurons in the VTA. **(I)** Left: Representative image (coronal plane, dashed midline, 100 μm, 20×) of LHb receiving cells in the VTA (green) and TH + neurons (violet). Right: Close ups showing degree of colocalization of GFP and TH (10 μm). **(J)** Cumulative probability distribution (KS test, *p* = 0.0063) for the entire population of counted cells of each section; TH + (black) and TH– (gray). Inset, total cell counts for individual mice (*n* = 4). MR, Median Raphe; ml, medial lemniscus; fr, fasciculus retroflexus; RAF, Relative Arbitrary Fluorescence units; ML, Medial-Lateral; AP, Anterior-Posterior; GFP, Green Fluorescence Protein; TH, Tyrosine Hydroxylase.

To examine which VTA territory LHb projections innervate, we firstly injected the LHb of mice with a rAAV8-tdTomato and then *i.* employed CLARITY to clear the brain in a whole-mount preparation, *ii.* registered the imaged volume from a mesoscale selective plane-illumination microscope and *iii.* examined resultant axonal arborization in the midbrain (see section “Materials and Methods”; Figures 1A,B) (Chung and Deisseroth, 2013; Voigt et al., 2019). Qualitative image analysis revealed dense LHb projections medially within midbrain structures as well as raphe nucleus (Figure 1B). Despite offering a global picture for the innervation stemming from the LHb, whole brain imaging fails in providing where LHb axons are making synapses within the midbrain. To identify LHb axons as well as their presynaptic terminals, we employed rAAV5-mRuby-T2A-Synaptophysin-eGFP injected into the LHb (Figure 1C). Long-range projecting LHb neurons densely innervated the midbrain with the majority of green punctae located in the medial territory of the VTA, and as expected by previous data also in the rostromedial tegmental nucleus [Figure 1D and Supplementary Figures 1A,B (Jhou et al., 2009)]. Accordingly, juxtacellular labeling of single LHb neurons in anesthetized mice and subsequent histological processing revealed LHb axonal processes and presynaptic boutons within medial VTA territories (Figures 1E–G).

Which neuronal populations are these LHb axons innervating? The medial VTA contains heterogeneous neuronal populations including dopamine releasing cells and non-dopaminergic neurons [i.e., GABA and glutamate containing (Morales and Margolis, 2017)]. To identify which VTA neurons receive LHb synaptic input we concomitantly injected an anterograde trans-synaptic high titer rAAV1:ht-Cre in the LHb and a cre-dependent rAAV8-Flex-EGFP in the VTA. This intersectional strategy, in combination with immunohistochemical staining for tyrosine hydroxylase (TH) permits the identification of those VTA neuronal populations (dopamine and non-dopamine producing) innervated by LHb axons (Figures 1H–J; Zingg et al., 2017). LHb-to-VTA neurons (GFP +) were predominantly negative to TH staining. Quantification showed that only 30.9 ± 2.9% of LHb receiving neurons in the VTA also stained for TH, whilst 32.3 ± 7.6% of total VTA TH + neurons were targeted by the LHb (21 sections/4 mice; Figure 1J). Altogether, this indicates that a minority of the LHb-to-VTA projection target TH + cells, rather controlling a neuronal population that is mostly TH– and located in the medial aspect of the VTA.

### Excitatory Synaptic Transmission Within the VTA During Cocaine Withdrawal

**FIGURE 2:**
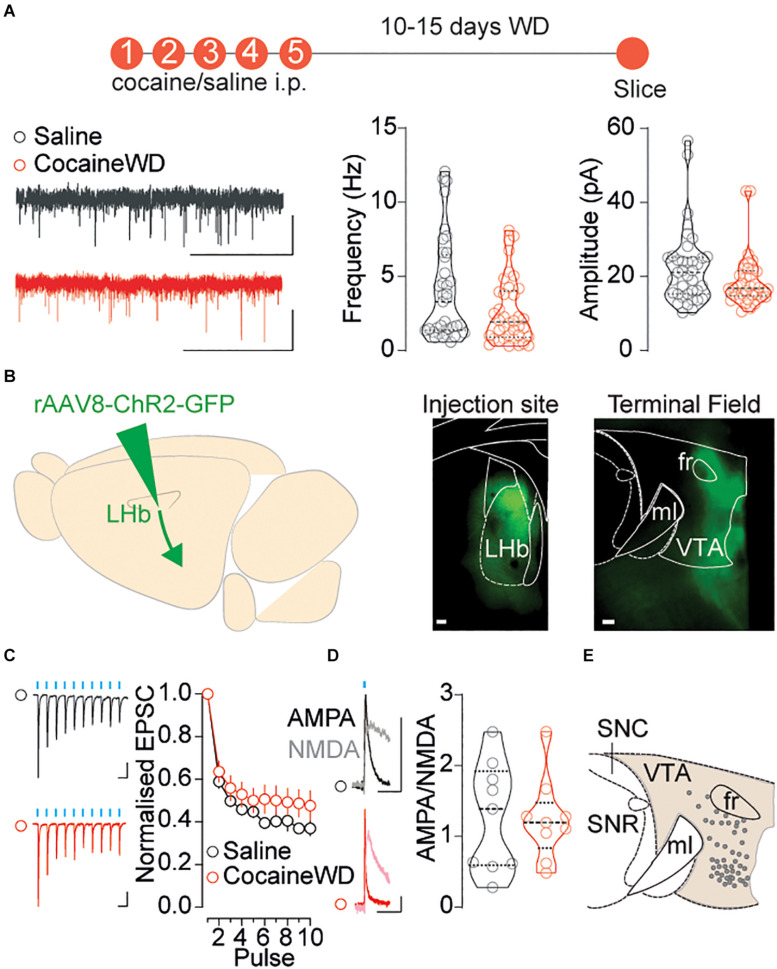
Examining excitatory synaptic transmission on to VTA neurons during cocaine withdrawal. **(A)** Top: Cocaine/Saline treatment protocol. Left: Sample sEPSCs traces (2s, 20pA) for saline (black) and cocaine treated (red) mice. Right: Violin plots for the frequency and amplitude of events in recorded cells (saline *n*_cells/mice_ = 37/16; cocaine *n*_cells/mice_ = 38/12; outlier cells_saline/cocaine_ = 5/6) Frequency Mann-Whitney, *U* = 531, *p* = 0.0690; Amplitude Mann-Whitney, *U* = 531, *p* = 0.0690. **(B)** Left: Viral strategy to investigate LHb innervation of VTA. Right: Injection site in the LHb and terminal field of GFP positive terminals in the VTA (both horizontal plane, 100 μm, 2.5×). **(C)** Sample traces (Saline: 50 ms, 100 pA; Cocaine: 50 ms, 20 pA) and normalized EPSC vs pulse plot (saline *n*_cells/mice_ = 27/10; cocaine *n*_cells/mice_ = 29/14) Interaction factor *F*(9,486) = 1.458, *P* = 0.1606 two-way ANOVA Repeated Measures. **(D)** Sample traces (50 ms, 20 pA) and violin plot showing LHb-to-VTA AMPA/NMDA of recorded cells (saline *n*_cells/mice_ = 9/4; cocaine *n*_cells/mice_ = 9/4) Two-sided *t*-test, *t*_16_ = 0.1022, *p* = 0.9199. **(E)** Map of recorded VTA cells. Data are presented as mean ± SEM. SNR, Substantia Nigra pars Reticulata; SNC, Substantia Nigra pars Compacta.

Glutamatergic transmission onto both LHb and VTA neuronal populations dynamically and through various mechanisms adapts during cocaine experience and withdrawal, subsequently underlying different addictive behavioral states (Meye et al., 2015; Morales and Margolis, 2017). Notably, LHb activity may, at least partly, influence the firing of VTA neurons (Matsumoto and Hikosaka, 2007). We therefore investigated whether cocaine withdrawal affects neurotransmission onto the VTA, and whether this relies on precise adaptations at LHb-to-VTA synapses. Firstly, we examined whether cocaine withdrawal alters excitatory transmission specifically onto the medial VTA by using a regime of five consecutive daily intraperitoneal injections of cocaine (20 mg/kg) followed by a 10–15 day withdrawal period. This specific protocol elicits increases in AMPA-receptors mediated neurotransmission, and leads to increased neuronal excitability within the LHb (Meye et al., 2015). As a consequence these adaptations lead to depressive-like symptoms in mice (Meye et al., 2015). First, we recorded spontaneous excitatory postsynaptic currents (sEPSCs) from neurons located within the medial territory of the VTA, and found that cocaine withdrawal did not alter overall frequency or amplitude of events (Figure 2A). To investigate more specifically how cocaine withdrawal may impact synaptic transmission at LHb-to-VTA synapses, we injected rAAV8-ChR2-GFP into the LHb and obtained whole-cell patch-clamp recordings from neurons in the medial VTA. Blue light stimulation of LHb terminals, in the presence of picrotoxin and APV to block GABA and NMDA receptors, respectively, evoked excitatory postsynaptic currents in virtually all medial VTA neurons (Figures 2B,C). Cocaine withdrawal did not significantly alter presynaptic neurotransmitter release probability inferred from the degree of frequency depression of EPSCs during a train of blue light pulses (10 pulses, 10 Hz, and 20 Hz) (Figure 2C and Supplementary Figures 2A–D). Similarly, cocaine withdrawal left unaffected optogenetically evoked AMPAR to NMDAR ratios (AMPAR/NMDAR, + 40 mV; Figures 2D,E) at LHb-to-VTA synapses. Hence, despite cocaine withdrawal increasing LHb neuronal excitability, it unexpectedly does not alter LHb excitatory transmission when macroscopically recording from medially located VTA cells.

### LHb Innervation of Output-Specific VTA Populations in Cocaine Withdrawal

**FIGURE 3:**
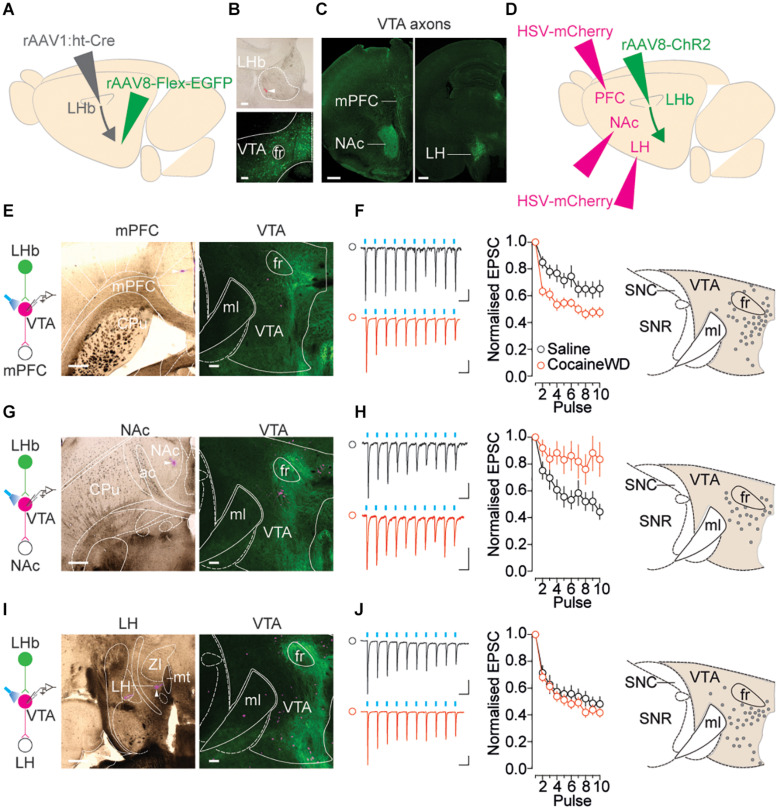
Cocaine withdrawal re-shapes glutamatergic transmission at LHb-to-VTA output circuits. **(A)** Left: Viral strategy to visualize projection targets of LHb-receiving VTA neurons. Right: Respective terminal fields in mPFC, NAc, and LH. **(B)** Top: Site of rAAV1:ht-Cre in the LHb (tip of injection needle indicated by white arrow and small deposit of red retrobeads). Bottom: LHb-receiving, GFP positive neurons in the VTA following rAAV8-Flex-GFP injection (both coronal plane, 100 μm, 10×). **(C)** Terminal zones of axonal projections of LHb receiving VTA neurons (both coronal plane, 500 μm, 2.5×). **(D)** Viral strategy to interrogate LHb innervation of output specific VTA populations. **(E)** Left: HSV-mCherry injection site in mPFC. Right: Overlapping LHb terminals (GFP) with retrogradely labeled VTA-to-mPFC neurons. **(F)** Left: Sample traces (Saline: 50 ms, 20 pA; Cocaine: 50 ms, 100 pA) and normalized EPSC vs. pulse plots (saline *n*_cells/mice_ = 21/16; cocaine *n*_cells/mice_ = 21/16) Interaction factor *F*(9,378) = 4.535, *P* < 0.0001 two-way ANOVA Repeated Measures. Right: map of recorded VTA-to-mPFC cells. **(G)** Left: HSV-mCherry injection site in NAc. Right: Overlapping LHb terminals with retrogradely labeled VTA-to-NAc neurons. **(H)** Left: Sample traces (Saline: 50 ms, 20 pA; Cocaine: 50 ms, 20 pA) and normalized EPSC vs. pulse plots (saline *n*_cells/mice_ = 11/5; cocaine *n*_cells/mice_ = 13/3) Interaction factor *F*(9,198) = 2.953, *P* = 0.0026 two-way ANOVA Repeated Measures. Right: map of recorded VTA-to-NAc cells. **(I)** Left: HSV-mCherry injection site in LH. Right: Overlapping LHb terminals with retrogradely labeled VTA-to-LH neurons. **(J)** Left: Sample traces (Saline: 50 ms, 50 pA; Cocaine: 50 ms, 100 pA) and normalized EPSC vs. pulse plots (saline *n*_cells/mice_ = 23/5; cocaine *n*_cells/mice_ = 24/6) Interaction factor *F*(9,405) = 0.7625, *P* = 0.6488 two-way ANOVA Repeated Measures. Right: map of recorded VTA-to-LH cells. For images of HSV injection sites in mPFC, NAc and LH – horizontal plane, 500 μm, 2.5×. For images of VTA in same mice – horizontal plane, scale bar, 100 μm, 5×. CPu, Caudate Putamen; ac, anterior commissure; ZI, Zona Incerta; mt, mammillothalamic tract.

Neurons within the VTA project their axons to a large variety of target structures – including mPFC and NAc – and shape rewarding and aversive behaviors in an output-specific fashion (Lammel et al., 2012). Therefore, the overall absence of cocaine withdrawal-driven synaptic changes does not rule out a scenario in which the LHb-to-VTA projection adapts according to the downstream target of VTA cells. To test this, we combined the high-titer anterograde trans-synaptic rAAV1:ht-Cre in LHb and cre-dependent EGFP expression in VTA to define the output circuit of LHb receiving VTA neurons (Figures 3A–C). Through serial section analysis we examined projections of LHb-innervated cells within the VTA. Terminal fields of these VTA projections were observed in mPFC, NAc and LH, structures vulnerable to cocaine experience (Lammel et al., 2014; Aransay et al., 2015). Next, a retrograde HSV-mCherry was injected either in the mPFC, NAc or LH concomitantly with a rAAV8-ChR2-GFP within the LHb (Figure 3D). A vast majority of retrogradely labeled neurons emerging from the different output structures were medially located within the VTA, and mingled within the mesh of LHb terminals (Figures 3E–I). Based on the evidence that drug withdrawal increases neuronal activity within the LHb, we examined LHb-driven presynaptic release onto these target-identified VTA populations (Meye et al., 2015, 2017). Blue light-evoked trains of presynaptic stimulation revealed that cocaine withdrawal increased presynaptic depression at LHb inputs onto VTA-to-mPFC, reduced presynaptic depression at LHb inputs onto VTA-to-NAc and left unaffected synaptic transmission onto VTA-to-LH projections (Figures 3F–J). This suggests that cocaine withdrawal produces presynaptic LHb glutamate release adaptations that depend on the projection target of the connected VTA neuron.

## Discussion

The negative behavioral state emerging during cocaine withdrawal can persist long after cessation of drug use and can involve symptoms reminiscent of depressive states (Meye et al., 2017). Dysfunction of LHb and VTA neurons contributes to the negative behavioral phenotypes emerging during drug withdrawal. However, the detailed neuronal circuits within the VTA that are under LHb control, and are gone awry during drug withdrawal, remain to be defined. Here, we provide evidence that LHb inputs to the VTA undergo diverse synaptic adaptations onto distinct VTA neuronal populations that project to different target structures.

The behavioral relevance of distinct neural populations often segregates based on their projection targets, a concept that especially applies to VTA circuits. Indeed, VTA populations and namely dopamine releasing cells underlie aversive or appetitive behaviors depending whether they project their axons to mPFC or precise territories of the NAc (Lammel et al., 2012; de Jong et al., 2019). In light of the LHb contribution to aversive states, it is plausible that LHb innervation would control VTA cells embedded in the aversion-related neuronal circuits. The intersectional approach using anterograde trans-synaptic labeling indicates that LHb provides input not only to TH + but also and preferentially to TH– neurons (Omelchenko et al., 2009). The axons of these cells reach, in turn, structures including the mPFC, the NAc and the LH, thereby defining trisynaptic circuits that are likely (mPFC, NAc), or not (LH), underlying aspects of aversive and cocaine-experience driven states (Meye et al., 2017; de Jong et al., 2019). One caveat of the anterograde trans-synaptic tracing is that it lacks cell specificity. The experimental conditions employed do not permit distinguishing the nature of the axonal projections (TH + or TH–). Therefore, future analysis to functionally dissect whether LHb-to-TH– VTA neurons controls local or long-range synaptic transmission is required to better define this intricate connectivity.

Cocaine withdrawal differentially alters presynaptic release of glutamate from the LHb to VTA neurons depending on their output target. The increased presynaptic depression at LHb to mPFC projecting VTA neurons is in line with a reported LHb increased neuronal activity during cocaine withdrawal (Meye et al., 2015). Accordingly, this trisynaptic circuit may underlie the negative states emerging in cocaine withdrawal, given the implications of VTA-to-mPFC in aversion and depressive like behaviors (Weele et al., 2019). On the other hand, the reduction in presynaptic glutamate release at LHb-VTA-NAc projections is a surprising result and diverges from the finding describing a cocaine withdrawal-mediated increase of LHb neuronal activity (Meye et al., 2015). Several scenarios may emerge that can explain this discrepancy: (i) that cocaine withdrawal differently affects neuronal activity amongst cell populations within the LHb, a scenario yet non-described in literature, or (ii) that cocaine withdrawal alters presynaptic release independently of somatic firing. Cocaine withdrawal seems inefficient in altering LHb excitatory synapses onto LH-projecting VTA neurons. However, we cannot rule out that opposing effects at TH + and TH– VTA neurons are taking place masking net effects. Finally, the LHb-controlled TH– population may in turn provide synaptic inhibition to neighboring dopamine neurons, highlighting the need of a better understanding of synaptic inhibition after cocaine withdrawal. Altogether, the use of refined genetic tools including cre driver mouse lines together with intersectional viral strategies and electrophysiology will be necessary to parse out these distinct mechanisms.

In conclusion, this study indicates that the LHb anatomically and functionally controls distinct subpopulations of VTA neurons that are in turn embedded in distinct circuits. The diverse forms of synaptic plasticity within these LHb-to-VTA circuits may contribute to shaping reward and aversion as well as behavioral phenotypes typical of drug withdrawal.

## Data Availability Statement

The original contributions presented in the study are included in the article/Supplementary Material, further inquiries can be directed to the corresponding author.

## Ethics Statement

The animal study was reviewed and approved by the Veterinary Offices of Vaud.

## Author Contributions

JC performed and analyzed immunostaining and electrophysiological experiments. IZ, PP-F, and AB performed *in vivo* juxtacellular labeling and anatomical analysis. AT, LB, and SP performed whole brain clarification, imaging, and analysis. FV conceived the MESO-Spim strategy. JC and MM conceptualized the study and wrote the manuscript with the help of all authors.

## Conflict of Interest

The authors declare that the research was conducted in the absence of any commercial or financial relationships that could be construed as a potential conflict of interest.

## Supplementary Material

Supplementary Figure 1 LHb terminal distribution in the RMTg. **(A)** Images (horizontal plane, Scale bar, 100 μm, 10×) left to right: injection site in the LHb; mRuby positive axons in the RMTg; Synaptophysin-EGFP positive terminals in the RMTg. **(B)** Quantification in Relative Arbitrary Fluorescent (RAF) Units of Synaptophysin expression throughout the mediolateral (ML, top) and Anterior-Posterior (AP, bottom) extent of the RMTg (*n*_hemispheres/mice_ = 3/2).

Supplementary Figure 2 10Hz stimulation of LHb axons on to defined VTA populations. **(A)** VTA global: Sample traces (Saline: 100 ms, 20 pA; Cocaine: 100 ms, 20 pA) and normalized EPSC vs. pulse plots (saline *n*_cells/mice_ = 27/10; cocaine *n*_cells/mice_ = 29/14) Interaction factor *F*(9,486) = 1.148, *P* = 0.3272 two-way ANOVA Repeated Measures. **(B)** VTA-to-mPFC: Sample traces (Saline: 100 ms, 20 pA; Cocaine: 100 ms, 20 pA) and normalized EPSC vs. pulse plots (saline *n*_cells/mice_ = 21/16; cocaine *n*_cells/mice_ = 21/16) Interaction factor *F*(9,162) = 2.141, *P* = 0.0289 two-way ANOVA Repeated Measures. **(C)** VTA-to-NAc: Sample traces (Saline: 100 ms, 20 pA; Cocaine: 100 ms, 10 pA) and normalized EPSC vs. pulse plots (saline *n*_cells/mice_ = 11/5; cocaine *n*_cells/mice_ = 13/3) Interaction factor *F*(9,198) = 2.012, *P* = 0.0397 two-way ANOVA Repeated Measures. **(D)** VTA-to-LH: Sample traces (Saline: 100 ms, 10 pA; Cocaine: 100 ms, 100 pA) and normalized EPSC vs. pulse plots (saline *n*_cells/mice_ = 23/5; cocaine *n*_cells/mice_ = 24/6) Interaction factor *F*(9,405) = 1.417, *P* = 0.1786 two-way ANOVA Repeated Measures.
