# Supplementary figures and images for: Output-Specific Adaptation of Habenula-Midbrain Excitatory Synapses During Cocaine Withdrawal
Source: Front Synaptic Neurosci. 2021 Mar 31;13:643138. doi: 10.3389/fnsyn.2021.643138 (PMC8044201; doi:10.3389/fnsyn.2021.643138)

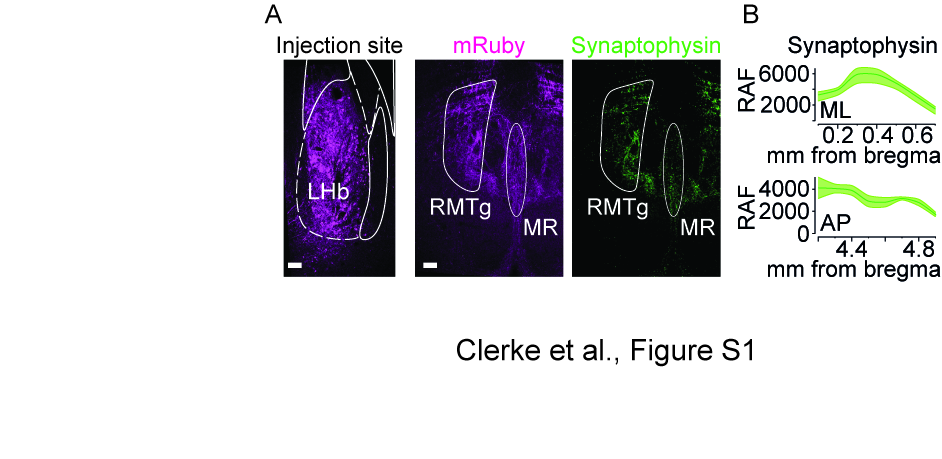

Supplement: Supplementary Figure 1 — LHb terminal distribution in the RMTg. (A) Images (horizontal plane, Scale bar, 100 μm, 10×) left to right: injection site in the LHb; mRuby positive axons in the RMTg; Synaptophysin-EGFP positive terminals in the RMTg. (B) Quantification in Relative Arbitrary Fluorescent (RAF) Units of Synaptophysin expression throughout the mediolateral (ML, top) and Anterior-Posterior (AP, bottom) extent of the RMTg (nhemispheres/mice = 3/2). [file Image_1.TIF]

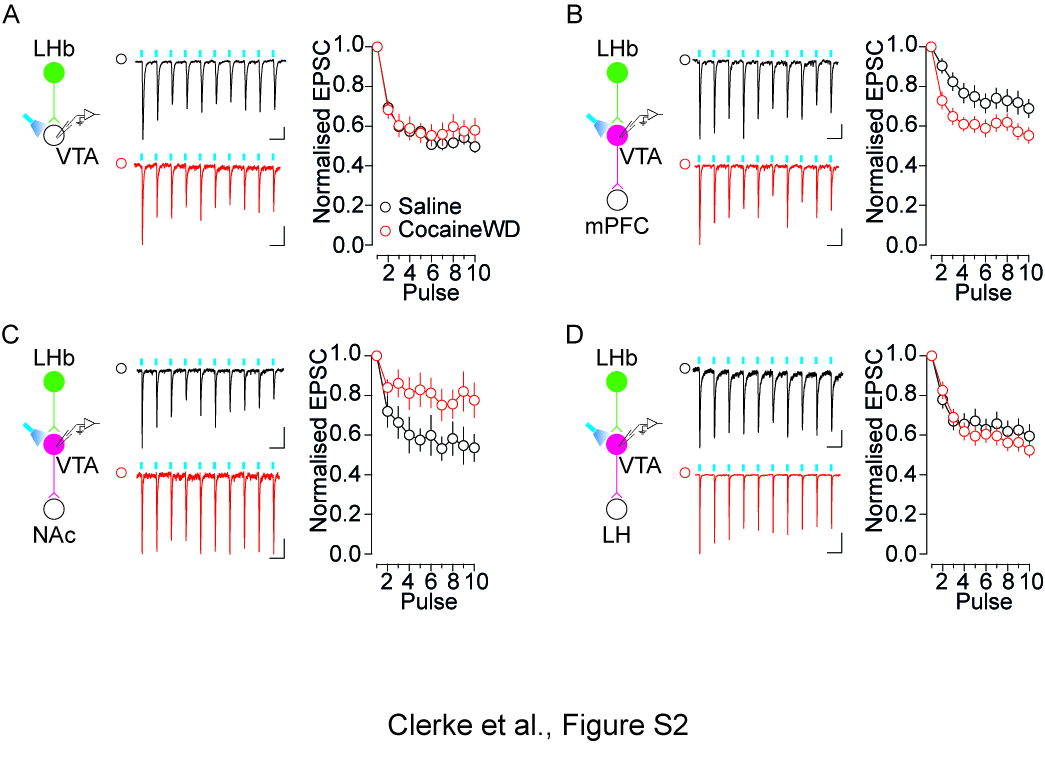

Supplement: Supplementary Figure 2 — 10Hz stimulation of LHb axons on to defined VTA populations. (A) VTA global: Sample traces (Saline: 100 ms, 20 pA; Cocaine: 100 ms, 20 pA) and normalized EPSC vs. pulse plots (saline ncells/mice = 27/10; cocaine ncells/mice = 29/14) Interaction factor F(9,486) = 1.148, P = 0.3272 two-way ANOVA Repeated Measures. (B) VTA-to-mPFC: Sample traces (Saline: 100 ms, 20 pA; Cocaine: 100 ms, 20 pA) and normalized EPSC vs. pulse plots (saline ncells/mice = 21/16; cocaine ncells/mice = 21/16) Interaction factor F(9,162) = 2.141, P = 0.0289 two-way ANOVA Repeated Measures. (C) VTA-to-NAc: Sample traces (Saline: 100 ms, 20 pA; Cocaine: 100 ms, 10 pA) and normalized EPSC vs. pulse plots (saline ncells/mice = 11/5; cocaine ncells/mice = 13/3) Interaction factor F(9,198) = 2.012, P = 0.0397 two-way ANOVA Repeated Measures. (D) VTA-to-LH: Sample traces (Saline: 100 ms, 10 pA; Cocaine: 100 ms, 100 pA) and normalized EPSC vs. pulse plots (saline ncells/mice = 23/5; cocaine ncells/mice = 24/6) Interaction factor F(9,405) = 1.417, P = 0.1786 two-way ANOVA Repeated Measures. [file Image_2.TIF]
